# Disparity in pseudohyphal morphogenic switching response to the quorum sensing molecule 2-phenylethanol in commercial brewing strains of *Saccharomyces cerevisiae*

**DOI:** 10.1093/femsmc/xtad002

**Published:** 2023-01-09

**Authors:** Scott J Britton, Lisa J Rogers, Jane S White, Hedwig Neven, Dawn L Maskell

**Affiliations:** International Centre for Brewing and Distilling, Institute of Biological Chemistry, Biophysics, and Bioengineering, School of Engineering and Physical Sciences, Heriot-Watt University, EH14 4AS Edinburgh, United Kingdom; Research & Development, Brewery Duvel Moortgat, 2870 Puurs-Sint-Amands, Belgium; TranscendED, Brooklyn, 11232 NY, United States; International Centre for Brewing and Distilling, Institute of Biological Chemistry, Biophysics, and Bioengineering, School of Engineering and Physical Sciences, Heriot-Watt University, EH14 4AS Edinburgh, United Kingdom; Research & Development, Brewery Duvel Moortgat, 2870 Puurs-Sint-Amands, Belgium; Department M2S, Centre for Food and Microbial Technology (CLMT), KU Leuven, 3000 Leuven, Belgium; International Centre for Brewing and Distilling, Institute of Biological Chemistry, Biophysics, and Bioengineering, School of Engineering and Physical Sciences, Heriot-Watt University, EH14 4AS Edinburgh, United Kingdom

**Keywords:** quorum sensing, filamentous growth, morphogenesis, aromatic alcohols, intercellular signaling, pseudohyphae

## Abstract

*Saccharomyces cerevisiae* can undergo filamentous growth in response to specific environmental stressors, particularly nitrogen-limitation, whereby cells undergo pseudohyphal differentiation, a process where cells transition from a singular ellipsoidal appearance to multicellular filamentous chains from the incomplete scission of the mother-daughter cells. Previously, it was demonstrated that filamentous growth in *S. cerevisiae* is co-regulated by multiple signaling networks, including the glucose-sensing RAS/cAMP-PKA and SNF pathways, the nutrient-sensing TOR pathway, the filamentous growth MAPK pathway, and the Rim101 pathway, and can be induced by quorum-sensing aromatic alcohols, such as 2-phenylethanol. However, the prevalent research on the yeast-pseudohyphal transition and its induction by aromatic alcohols in *S. cerevisiae* has been primarily limited to the strain Σ1278b. Due to the prospective influence of quorum sensing on commercial fermentation, the native variation of yeast-to-filamentous phenotypic transition and its induction by 2-phenylethanol in commercial brewing strains was investigated. Image analysis software was exploited to enumerate the magnitude of whole colony filamentation in 16 commercial strains cultured on nitrogen-limiting SLAD medium; some supplemented with exogenous 2-phenylethanol. The results demonstrate that phenotypic switching is a generalized, highly varied response occurring only in select brewing strains. Nevertheless, strains exhibiting switching behavior altered their filamentation response to exogenous concentrations of 2-phenylethanol.

## Teaser

Variation observed in filamentous growth response to quorum-sensing compound 2-phenylethanol in commercial isolates of Saccharomyces cerevisiae.

## Introduction

Microorganisms have evolved various intercellular communication mechanisms to benefit community fitness within the external milieu (West et al. 2006, Smukalla et al. 2008, Darch et al. 2012, Özkaya et al. 2017, Combarnous and Nguyen 2020). Quorum sensing (QS) is the most well-studied of these known mechanisms. QS communication relies on the diffusion of small hormone-like signaling molecules, known as autoinducers (AI) or quorum-sensing molecules (QSMs), to synchronize community-wide gene expression in tandem with population density (West et al. 2006, Albuquerque and Casadevall 2012, Bandara et al. 2012, Britton et al. 2021). As QSMs are secreted and accumulate throughout the growth phase, the QSM triggers synchronized gene expression by binding to its cognate receptor to produce a coordinated response upon reaching a threshold concentration (Bassler 2002, Waters and Bassler 2005).

QS gene regulation was described initially in the late 1960s in the Gram-negative bacterium *Aliivibrio fischeri*, where inoculating cells into a previously ‘conditioned’ growth medium led to the transcriptional activation of the bacterial bioluminescent system (Nealson et al. 1970). In this case, transcription of the bacterial luciferase gene (lux) was later shown to be governed by above threshold concentrations of the low-molecular-weight acyl-homoserine lactone (Eberhard et al. 1981). Subsequently, for decades, bacterial QS has been extensively examined and shown to regulate diverse cellular processes, including bioluminescence (Engebrecht and Silverman 1984); competence and sporulation (Rai et al. 2015); social motility (Quiñones et al. 2005, Nickzad et al. 2015); virulence (Quiñones et al. 2005, Ali et al. 2017); antibiotic production (Liu et al. 2007); stress adaptation (García-Contreras et al. 2015); cell morphology and dimorphism (Rai et al. 2015); nutrient uptake (An et al. 2014); and more.

Although the knowledge about QS regulation in fungi is far more limited in comparison, a substantial number of investigations have focused on the role of fungi QS regulation on the dimorphic switching response, including the earliest report of cell-density dependent regulation in fungi describing the regulation of filamentation in the human fungal pathogen *Candida albicans* in 1969 (Lingappa et al. 1968, Britton et al. 2021). Over 40 years later, Hornby et al. (2001) identified the farnesane sesquiterpenoid, farnesol, as the QS molecule chiefly responsible for governing this fungal-specific yeast-to-hyphal transition response. The yeast-to-hyphal transition has been shown to play a significant role in fungal host–cell attachment, evasion from host defenses, and facilitating tissue invasion; where it has been shown that the mechanical forces associated with hyphal growth in some fungi were sufficient enough to elicit cell damage and penetration into epithelial cells (Rooney and Klein 2002, Moyes et al. 2016, Westman et al. 2019). As filamentous growth is entwined with QS and virulence across many fungi, the phenotypic switch between yeast and filamentous forms has become the primary focus of contemporary virulence research. However, as not all fungi are well characterized, *Saccharomyces cerevisiae* is often exploited to study numerous biological processes, including yeast-to-filamentous transition, due to its genetic tractability and analogous signaling pathways.

In *S. cerevisiae*, Chen & Fink were the first to demonstrate that conditioned supernatant and the aromatic alcohol 2-phenylethanol (2-PE) induced filamentation, in laboratory strain Σ1278b, under restrictive nitrogen conditions (Chen and Fink 2006). This phenotypic transition is often viewed as an analogous adaptive response to motility, where the reorganization of polarity, increase in cell length, and incomplete scission facilitates the foraging for available nutrients (Gimeno et al. 1992, Kron 1997, Gancedo 2001, Hornby et al. 2001, Chen et al. 2004, Nickerson et al. 2006, Cullen and Sprague 2012). Genomic analysis has thus far revealed five evolutionarily conserved signaling pathways involved in this nutrient-induced developmental response: (i) the cAMP-PKA pathway; (ii) the TOR pathway; (iii) the SNF1/AMPK pathway; (iv) the Rim101 pathway; and (v) the Kss1-MAPK pathway (Roberts and Fink 1994, Ceccato-Antonini 2008, Granek et al. 2011, Cullen and Sprague 2012, Ryan et al. 2012). These pleiotropic signaling pathways play a role in the transcriptional expression of *FLO11*, previously designated as *MUC1*, encoding a cell surface flocculin protein with a structure comparable to yeast serine/threonine-rich glycosylphosphatidylinositol(GPI)-anchored cell wall proteins (Lo and Dranginis 1998, Zara et al. 2009). These specialized cell-surface proteins are known for their involvement in filamentation; surface adhesion; biofilm/mat formation; velum development; and flocculation, an asexual calcium-dependent form of cell-cell aggregation (Lo and Dranginis 1998, Bayly et al. 2005, Fidalgo et al. 2006, Zara et al. 2009, Soares 2011, Andersen 2014, Yang et al. 2018, Chow et al. 2019, Bouyx et al. 2021, Huismann et al. 2021).

Until recently, it was long held that the filamentation response to quorum sensing molecule 2-PE was prevalent throughout the *S. cerevisiae* population. However, a study by Lenhart et. al. (2019) demonstrated that naturally isolated strains did not induce filamentation in response to aromatic alcohols, suggesting QS-dependent filamentation may be strain-specific or less profuse than what was initially described. Nevertheless, the variation in the yeast-to-filament growth response in commercially relevant industrial strains remains under-explored, as the laboratory strain Σ1278b dominates studies related to this phenotype. Given that laboratory strains are seldomly genetically and phenotypically representative of wild-type strains found in nature or used commercially (Warringer et al. 2011), investigations on industrially relevant strains broaden the current understanding of quorum sensing strain variation within the wider *S. cerevisiae* population and its potential relevance to industrial applications.

A longstanding industrial application for *S. cerevisiae*, dating back thousands of years, is beer brewing (Lodolo et al. 2008, Sicard and Legras 2011). Within this process, *S. cerevisiae* is primarily inoculated into wort consisting of water, malted cereal grains, and hops. As a result, ethanol production occurs via the Embden-Meyerhof-Parnas pathway, which converts glucose to pyruvate and converts pyruvate to the primary by-products ethanol and carbon dioxide (Iorizzo et al. 2021). However, simultaneous production of secondary metabolites and auxiliary metabolic activity significantly influences the taste and aroma (Pires et al. 2014, Wauters et al. 2021). Assumption that the FLO11-encoded flocculins, governed by QS, are also responsible for the flocculent phenotype affecting yeast brewing performance, impacting fermentation efficiency and flavor generation, means that further investigation into the potential impact of QS on commercial brewing yeast is certainly warranted (Stewart 2018, Britton et al. 2021).

Here, the strain-specific phenotypic variability in pseudohyphal morphogenic switching response across 16 industrially relevant commercial brewing strains was investigated. Industrial yeast strains were cultured on SLAD medium [0.68% yeast nitrogen base w/o amino acids or ammonium sulfate, 2% dextrose, 50 µM ammonium sulfate, and 2% washed agar] supplemented with or without exogenous 2-phenylethanol at 30°C. Afterward; whole colony filamentation was quantified using an automated two-dimensional whole colony image analysis tool. Overall, our findings indicate a significant amount of strain-specific variation in the quorum sensing-dependent pseudohyphal switching response between commercially relevant brewing strains of *S. cerevisiae* and the yeast-to-filament morphogenic response to aromatic alcohols may be a more limited, strain-specific effect. Moreover, these results emphasize the importance of incorporating strains with varied genetic and ecological backgrounds when investigating biological responses.

## Materials and methods

### Strains

Investigations were carried out on 16 strains of *Saccharomyces cerevisiae* obtained from the yeast collections of White Labs (San Diego, California, USA), Fermentis (Marquette-lez-Lille, FR), Wyeast (Hood River, Oregon, USA), Imperial Yeast (Portland, Oregon, USA), and Duvel Moortgat, NV (Puurs-Sint-Amands, BE). Per manufacturer instructions, taxonomic identities were verified by real-time multiplex PCR (GEN-IAL, Troisdorf, Germany, QTPYB0096).

### Media

Yeast strains were pre-cultured in a liquid broth [YPD; 1% yeast extract, 2% peptone, and 2% dextrose]. Filamentous growth was induced on 4X synthetic low-ammonium dextrose agar [SLAD agar; 0.68% yeast nitrogen base w/o amino acids or ammonium sulfate, 2% dextrose (Biowest, Nuaillé, FR), 50 µM ammonium sulfate, and 2% washed agar (WVR International, Radnor, PA, USA] and when applicable, supplemented with 2-phenylethanol (Alfa Aesar, A15241) to a concentration of 100 µM or 200 µM just prior to plate pouring. Unless otherwise indicated, all materials were obtained from Sigma Aldrich, St. Louis, MO, USA.

### Filamentous growth assay

*Saccharomyces cerevisiae* strains were pre-cultured in YPD at 24 ± 2°C for 24 h. Cultures underwent serial dilution in 0.85% sterile saline and were inoculated to a 10^−5^ concentration on SLAD agar plates. For experimental conditions, SLAD was supplemented with 100 µM or 200 µM exogenous 2-PE. Plates were sealed with parafilm and incubated inverted for three days at 30°C. Following incubation, 10–15 colonies were randomly selected and photographed from each dish at 2.5 X total magnification using a ZEISS Axio Lab.A1 FL-LED microscope equipped with a ZEISS Axiocam 105 color camera (2.2 μm pixel resolution) and saved as a JPEG image. The assay was carried out in triplicate per condition, whereafter, ten whole colony photographs from each assay condition (n = 30) were chosen randomly selected for further image analysis and processing (n = 540).

### Image analysis and processing pipeline

**Figure 1.**
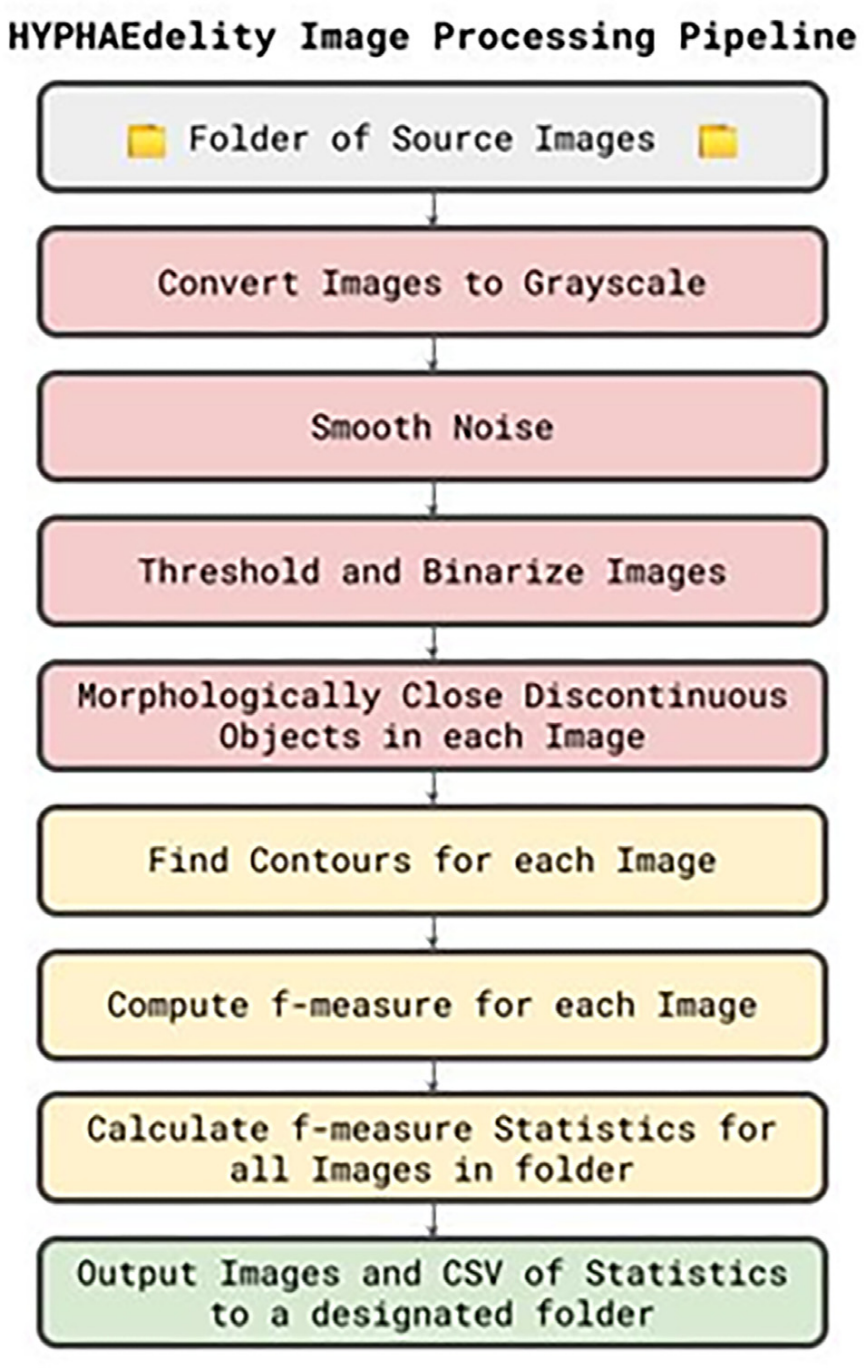
An schematic overview of the HYPHAEdelity image processing pipeline.

Image analysis and processing were completed using HYPHAEdelity (Britton et al. 2022) (Brooklyn, USA), the publicly available two-dimensional whole colony filamentation measurement tool utilizing the standard threshold setting. Uploaded JPEG images were automatically saved as a uniform resolution (2560×1920), converted from blue-green-red (BGR) to grayscale, smoothed with a Gaussian blur filter with a 5×5 rectangular kernel, converted to binary, and had small voids, resulting from overlapping filaments and filaments returning to the central colony mass, eliminated. Then the two largest contours present in each image account for (i) the boundary of all of the filamentous protrusions outside the central colony mass (referred to as A*_outer_*) and (ii) the boundary of the central colony mass (referred to as A*_inner_*) were determined. The total filamentous growth area, referred to as the *f-measure*, was computed by subtracting A*_inner_* from A*_outer_*. A schematic overview of the HYPHAEdelity image processing pipeline is seen in Fig. 1.

## Results

**Figure 2.**
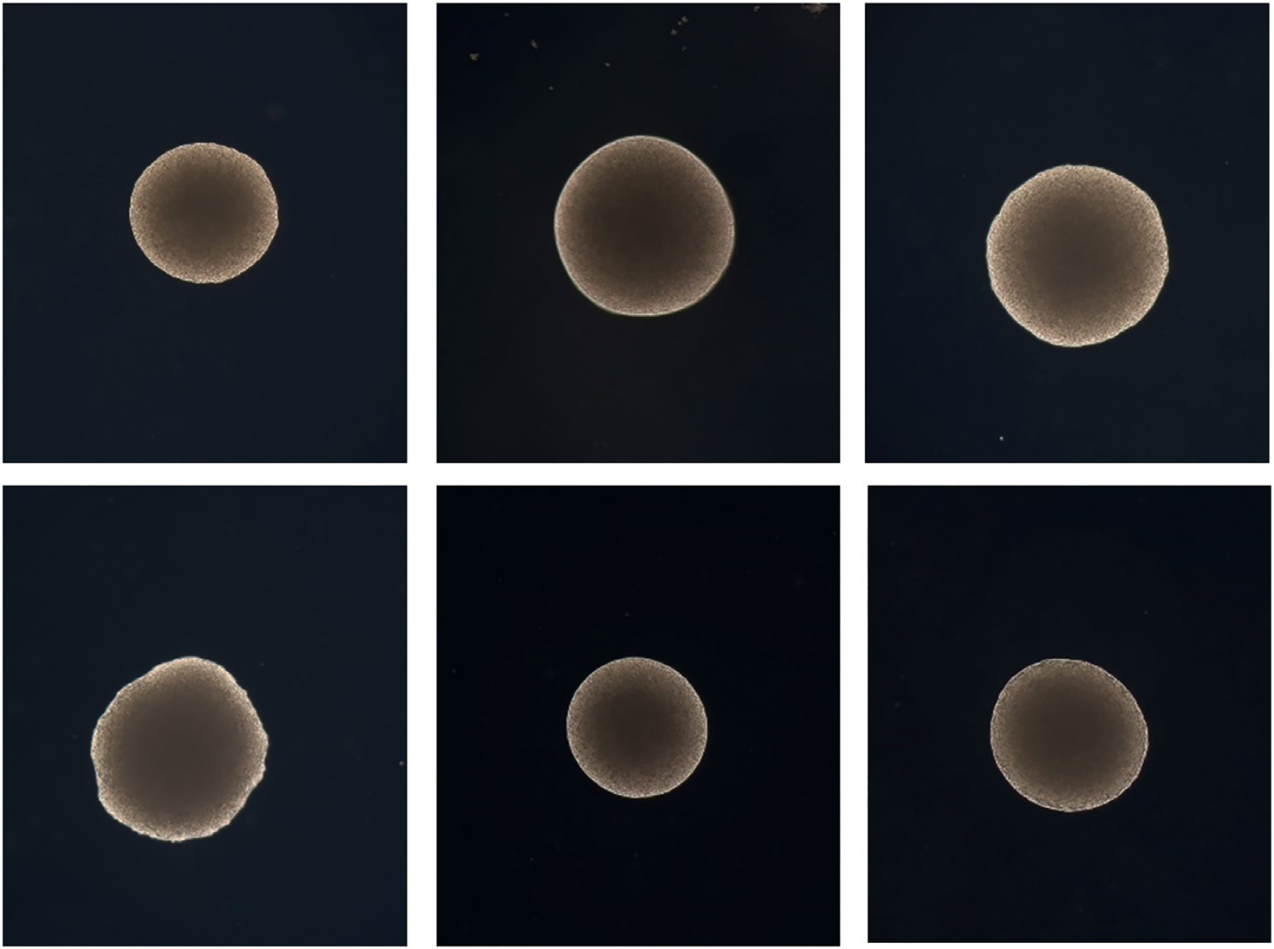
YMB4531 (top) and YMB4548 (bottom) at 0 µM (left), 100 µM (middle), 200 µM (right) 2-PE concentrations.

A Kruskal–Wallis test was executed to detect intra-strain *f-measures* differences between 0, 100, and 200 µM conditions following the removal of outliers via the Iglewicz and Hoaglin outlier test employing modified z-scores. This statistical method was selected as the distribution of *f-measures* was not similar between experimental groups. The χ^2^(3) statistics and *P* values for each strain are indicated within Table 1. YMB4519, YMD4521, YMD4537, YMD4538, YMD4541, YMD4545, YMD4552, and YMD4553 demonstrated a statistically significant difference (*P* < 0.05) in whole colony filamentation between 2-PE experimental groups. In comparison, YMD4531 and YMD4548 did not yield any significant difference (*P* < 0.05) between the 2-PE exposure groups. Moreover, YMD4525, YMD4529, YMD4533, YMD4534, YMD4542, and YMD4544 did not exhibit any whole colony filamentation employing visual assessment under any experimental condition and thus were not included in the statistical analysis (Fig. 2).

**Table 1.** Kruskal Wallis χ^2^(3) and *P*-value results comparing the intra-strain *f-measures* of commercial brewing strains grown on SLAD medium, supplemented with 0, 100, or 200 µM exogenous 2-PE, for 3-days at 30°C. N.D. (blue) indicates strains that failed to exhibit filamentation under any experimental condition and were not included in the statistical analysis.

Strain ID	Source Collection	χ^2^(3)	*P*-value
YMD4519	White Labs	10.751	0.005*
YMD4521	Duvel Moortgat	7.031	0.030*
YMD4525	Imperial Yeast	N.D.	N.D.
YMD4529	White Labs	N.D.	N.D.
YMD4531	Duvel Moortgat	2.044	0.360
YMD4533	White Labs	N.D.	N.D.
YMD4534	White Labs	N.D.	N.D.
YMD4537	Duvel Moortgat	13.661	0.001*
YMD4538	Fermentis	34.149	0.000*
YMD4541	White Labs	11.820	0.003*
YMD4542	White Labs	N.D.	N.D.
YMD4544	Duvel Moortgat	N.D.	N.D.
YMD4545	Duvel Moortgat	53.449	0.000*
YMD4548	White Labs	3.817	0.148
YMD4552	White Labs	13.203	0.001*
YMD4553	Wyeast	7.480	0.024*

* indicated statistically significant values (*α* = 0.05).

**Figure 3.**
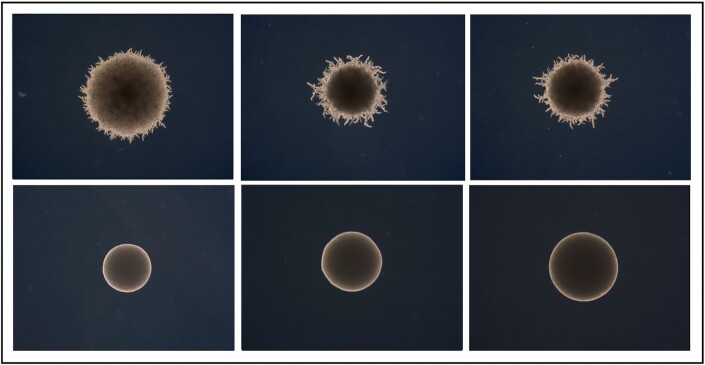
YMB4537 (top) and YMB 4544 (bottom) whole colonies filamentation at 0 µM (left), 100 µM (middle), 200 µM (right) 2-PE concentrations. YMB4537 visually demonstrates a significant change in filamentation degree resulting from 2-PE exposure, while YMB4544 exhibits no change.

Pairwise comparisons were performed on commercial strains demonstrating statistically significant differences (*P* < 0.05) between the means using the Dunn's test procedure with a Bonferroni correction for multiple comparisons (Table 2). Post hoc analysis revealed statistically significant differences (*P* < 0.05) in *f-measures* between 0 and 100 µM 2-PE exposure groups for strains YMD4519, YMD4537, YMD4538, YMD4541, YMD4545, and YMD4553 (Fig. 3). Statistically discernable (*P* < 0.05) differences in *f-measures* were further observed between 0 µM and 200 µM 2-PE exposure groups for YMD4538, YMD4545, and YMD4552, and between 100 and 200 µM 2-PE exposure groups for YMD4519, YMD4521, YMD4541, YMD4545, and YMD4552.

**Table 2.** Dunn's test with a Bonferroni correction for multiple comparisons between 0, 100, and 200 µM 2-PE treatment groups.

Strain ID	*P* 0/100 μM	*P* 0/200 μM	*P* 100/200 μM
YMD4519	0.018*	1	0.010*
YMD4521	1	0.106	0.043*
YMD4537	0.003*	1	0.007*
YMD4538	0.000*	0.000*	1
YMD4541	0.043*	1	0.003*
YMD4545	0.000*	0.000*	1
YMD4552	1	0.013*	0.002*
YMD4553	0.023*	0.198	1

* indicated statistically significant values (*α* = 0.05).

Pairwise comparisons of relative change between mean *f-measures* were calculated between 0/100 and 100 µM/200 µM experimental groups. Of the eight strains compared, YMD4537, YMD4538, YMD4541, and YMD4553 demonstrated a significant increase in filamentation, ranging from 12.47% to 113.60%, resulting from the 100 µM 2-PE treatment compared to the control. In contrast, YMD4519 and YMD4545 demonstrated a significant reduction, 2.63% and 77.16%, respectively, in filamentation under the same conditions (Table 3). Moreover, in the pairwise *f-measure* comparison between the 100 µM/200 µM experimental groups, most strains demonstrated an overall reduction in filamentation due to the increased concentration of 2-PE. However, one strain, YMD4519, showed a slight filamentation increase at 200 µM 2-PE concentrations.

**Table 3.** Pairwise comparison of relative change in *f-measure* means between the 0/100 and 0/200 µM experimental groups.

Strain ID	Pairwise Mean *f-measure* Comparison 0/100 µM	Pairwise Mean *f-measure* Comparison 100/200 µM
YMD4519	−2.63%	0.74%
YMD4521	–	−32.37%
YMD4537	33.17%	−23.17%
YMD4538	113.60%	–
YMD4541	12.47%	−23.36%
YMD4545	−77.16%	–
YMD4552	–	−30.90%
YMD4553	71.60%	–

## Discussion

Microbes, including fungi, can engage in various social phenotypes that provide fitness benefits to singular cells and genetic lines (West et al. 2006, Darch et al. 2012). Previously, the filamentous growth response was demonstrated in laboratory strain Σ1278b and, until recently, it was held (Lenhart et al. 2019) that this response was conserved across *Saccharomyces* spp (Kayikci and Magwene 2018) and other medically crucial fungal species (Wongsuk et al. 2016, Chow et al. 2019). Given the frequent genetic and phenotypic discrepancies observed between laboratory and wild-type strains (Bonhivers et al. 1998, Palkova 2004, Warringer et al. 2011), we aimed to assess the variation in the filamentous growth in response to 2-PE across a selection of brewing strains stemming from various geographical backgrounds given its potential relevance to yeast performance within the brewing industry (Britton et al. 2021).

A remarkable degree of phenotypic variation was observed between the 16 commercial strains cultured on nitrogen-restrictive media, with some strains displaying exceptionally pronounced filamentation. In contrast, others demonstrated no signs of filamentous growth when cultured under restrictive nitrogen conditions. A comparison between strains within the 0 µM condition suggests the filamentous growth response is not a widespread phenotype observed across all *S. cerevisiae* strains but rather a strain-specific variable response. Our results show that nearly 38% of the commercial yeast strains examined did not exhibit any indications of filamentation. In contrast, the remaining strains YMD4519, YMD4521, YMD4531, YMD4537, YMD4538, YMD4541, YMD4545, YMD4548, YMD4552, and YM4553 demonstrated varying degrees of whole colony filamentation, with *f-measures* ranging from 0.310 ± 0.199 to 0.965 ± 0.544 within the 0 μM treatment (Table 4). It is conceivable that culturing the non-filamentous strains under more severe nitrogen limitations could yield a filamentation response; however, we aimed to assay a general, robust response under nitrogen-limiting conditions and YMD4525, YMD4529, YMD4533, YMD4534, YMD4542, and YMD4544 did not. Moreover, the additional manual scrutiny of ‘non-filamentous’ whole colonies was undertaken and confirmed the complete absence of filament development within these colonies.

**Table 4.** Measurements of mean ± SD *f-measures* for strains demonstrating filamentation cultured on SLAD medium supplemented with 0, 100, or 200 μM 2-phenylethanol.

Strain ID	0 μM mean *f-measure* ± SD	100 μM mean *f-measure* ± SD	200 μM mean *f-measure* ± SD
YMD4519	0.324 ± 0.149	0.315 ± 0.398	0.318 ± 0.150
YMD4521	0.370 ± 0.141	0.427 ± 0.245	0.289 ± 0.145
YMD4531	0.364 ± 0.109	0.465 ± 0.271	0.381 ± 0.178
YMD4537	0.533 ± 0.138	0.710 ± 0.376	0.545 ± 0.163
YMD4538	0.689 ± 0.243	1.472 ± 0.836	1.074 ± 0.257
YMD4541	0.228 ± 0.108	0.257 ± 0.079	0.197 ± 0.056
YMD4545	0.965 ± 0.544	0.220 ± 0.090	0.213 ± 0.048
YMD4548	0.426 ± 0.978	0.624 ± 1.545	0.283 ± 0.598
YMD4552	0.310 ± 0.199	0.288 ± 0.137	0.199 ± 0.054
YMD4553	0.252 ± 0.142	0.433 ± 0.493	0.794 ± 1.421

Due to the limited number of strains analyzed and the absence of in-depth genomic analysis, it is impossible to discern whether the lack of filamentous response is associated with a particular phylogenetic clade. Although the yeasts included in this study come from diverse geographical backgrounds, comparative genomics has demonstrated that industrial yeasts stem from only a limited number of ancestral strains that later evolved into separate lineages, so despite their distant geographical origin, the domestication of beer yeast led closely related strains to be become dispersed geographically due to human-assisted redistribution (Gallone et al. 2016). Gallone et al.(2016) confirm this notion by demonstrating that beer yeasts in the United States are closely related to British beer yeasts, suggesting that they were selectively imported rather than originating from indigenous wild yeast populations. Another possible explanation is that the filamentation defect within these strains could result from an unrelated downstream mutation preventing the cellular expression of FLO11, despite the cell's capability to sense the quorum-sensing signal.

Generally, our results are consistent with the most recent findings by Lenhart et al. (2019) and Winters et al. (2022a), which confirmed the association between elevated concentrations of 2-phenylethanol and the induction of unipolar budding and the pseudohyphal filamentation. Moreover, our results also corroborate the additional conclusions of Lenhart et al., whereby the phenotypic switching is more a generalized response and strain specific.

However, we disagree with the conclusions proposed by Winters et al. (2022a), stating that 2-PE-induced filamentous growth cannot be deemed a quorum sensing mechanism as it does not meet the ‘physiological concentration’ requirement previously defined by Winters et al. (2019, 2021, 2022a). This conclusion was founded on their recent study demonstrating physiological concentrations to be 4.3 ± 0.4 μM following 30 hours of culture in a defined medium with a standard laboratory strain. Prior studies by Zupan et al. (2013) and Richter et al. (2013) demonstrated natural concentrations of 2-PE from *S. cerevisiae* fermentations originating from the wine industry, which were in some cases more than 100x the concentration reported by Winters et al.(2022b). Similarly, higher concentrations were also observed following the analysis of finished beers produced from industrial *S. cerevisiae* strains (Ayrapaa 1961, Li et al. 2021). Given that the biosynthesis of 2-PE has been demonstrated to be strain specific (Richter et al. 2013, Mitri et al. 2022), the general conclusion proposed by Winters et al. (2022b) suggests that 2-PE-induced filamentation in *S. cerevisiae* does not meet the definition of intercellular signaling mechanism due to the responsive mechanism only occurring beyond physiological concentrations; however, the physiological concentrations reported by Winters et al. (2022b) are not reflective of the natural occurrence observed within diffusion-limited fermentation environments employing industrially relevant strains.

Moreover, our results further demonstrate that the aromatic alcohol, 2-PE, is a critical quorum-sensing molecule capable of modulating the strain-specific phenotypic transition between unicellular yeast and the filamentous form of *S. cerevisiae*, as the exogenous supplementation of 2-PE influenced the magnitude of filamentation in YMD4519, YMD4521, YMD4537, YMD4538, YMD4541, YMD4545, YMD4552, and YMD4553 (Table 3). Although, it is not known why industrial strains YMD4537, YMD4538, YMD4541, and YMD4553 significantly increased filamentation at concentrations of 100 μM, while the opposite was observed in YMD4519 and YMD4545 (Table 3). Moreover, it is also not understood why some strains did not demonstrate any change in filamentation at the 100 μM concentration. This observation further supports that the stimulation of filamentous growth is likely a strain-specific response. However, elevating the exogenous concentration of 2-PE to 200 μM decreased the magnitude of filamentation (*f-measures*) in YMB4521, YMD4537, YMD4541, and YMD4552. Although it has been suggested that aromatic alcohol is cytotoxic at these high concentrations (Winters et al. 2022b), other research has demonstrated minor inhibitory effects when grown on a medium containing 1 g/L 2-PE (Jin et al. 2018).

While this research demonstrates that the 2-PE-induced filamentation response is strain-specific among commercial brewing strains, additional research is required to fully understand the full impact 2-PE may have on global gene expression, cell physiology, and the metabolic activity of industrially relevant *S. cerevisiae* and its relevance to the brewing industry. Further examinations into 2-PE induced gene expression could reveal QS circuits governing cellular processes that may positively or negatively impact commercial brewing activities, such as those linked to fermentation rate, the efficiency of ethanol production, *STA1* expression, ester formation, propagation, yeast vitality, flocculation, and the overall quality of the finished product.

Prior studies have already demonstrated that QS mechanisms exist in fermented foods (Johansen and Jespersen 2017) and have suggested that the regulation of inherent QS systems could enhance the quality of many fermented foods. Therefore, an increased understanding of QS-induced expression could potentially improve the overall quality and efficiency of alcoholic beverage production.

## Conclusion

These results demonstrate that pseudohyphal switching is a generalized, highly varied response occurring only in select commercial strains of *S. cerevisiae*. These observations corroborate previous findings observed in natural populations (Lenhart et al. 2019). However, what remains to be firmly understood is why some strains undergo morphogenesis in response to exogenous exposure to QS molecule 2-PE, while others do not. It is feasible that multiple rationales exist to explain this disparity across commercial brewing strains, as in (i) some strains may unable to sense 2-PE, while (ii) others may sense 2-PE, but be unable to initiate a response, and (iii) some strains may be unable to undergo filamentation. Although we did did not investigate these possibilities in this study, certainly the genetics underpinning the disparity in pseudohyphal switching response and the influence of 2-PE on gene expression in commerical strains of *S. cerevisiae* should be addressed in future work.
